# Supplementary material for: Integrative species delimitation based on COI, ITS, and morphological evidence illustrates a unique evolutionary history of the genus Paracercion (Odonata: Coenagrionidae)
Source: PeerJ. 2021 May 26;9:e11459. doi: 10.7717/peerj.11459 (PMC8164416; doi:10.7717/peerj.11459)
Supplement: Supplemental Information 4 [file peerj-09-11459-s004.docx]

**Table S1 Detailed collecting information of the specimens and the accession numbers of downloaded sequences in this study.**

| Species | Longitude | Latitude | Number | Location | Collector | Date | Altitude |
| --- | --- | --- | --- | --- | --- | --- | --- |
| *Paracercion ambiguum* | 106.348129 | 22.017735 | 2 | Huu Lien National Park, Northern Vietnam | Tom Kompier | 2013/11/23-12/1 |  |
| *Paracercion barbatum* | 103.421292 | 24.714277 | 34 | Changhu town, Shilin, Yunnan, China | Xin Ning | 2016/6/30 |  |
| *Paracercion calamorum* | 112.943373 | 28.194670 | 1 | Changsha, Hunan, China | Xin Ning | 2015/8/5 | 155 |
| *Paracercion calamorum* | 108.140086 | 25.066091 | 2 | Hechi, Guangxi, China | Xin Yu | 2005/7/22 | 226 |
| *Paracercion calamorum* | 128.919244 | 43.883723 | 11 | Ningan, Heilongjiang, China | Xin Ning | 2016/8/8 |  |
| *Paracercion calamorum* | 102.377088 | 25.064150 | 4 | Qinglong town, Yunnan, China | Xin Ning | 2016/6/28 |  |
| *Paracercion calamorum* | 117.178400 | 36.067000 | 2 | Taian, Shandong, China | Junli Xue | 2015/8/17 | 106 |
| *Paracercion calamorum* | 108.040358 | 25.240198 | 7 | Tianjin University, Tianjin, China | Xin Ning | 2015/6/13 |  |
| *Paracercion calamorum* | 121.583333 | 24.750000 | 1 | Yilan County, Taiwan, China | Qiang Xie | 2011/6/11 | 670 |
| *Paracercion calamorum* | 120.501065 | 29.543737 | 3 | Zhuji, Zhejiang, China | Xin Yu | 2013/4/23 |  |
| *Paracercion dorothea* | 99.742010 | 27.915062 | 6 | Shangri-La, Yunnan, China | Xin Yu | 2011/7/16-17 |  |
| *Paracercion hieroglyphicum* | 100.981277 | 41.827175 | 1 | Alxa League, Inner Mongolia, China | Yang Wang | 2016/7/25 |  |
| *Paracercion hieroglyphicum* | 106.043007 | 38.614936 | 4 | Helan Mountain, Ningxia, China |  | 2010/8/3 |  |
| *Paracercion hieroglyphicum* | 112.699768 | 40.578453 | 2 | Liangcheng County, Inner Mongolia, China | Wenbo Yi | 20130723 | 1212 |
| *Paracercion hieroglyphicum* | 129.638976 | 44.558647 | 5 | Mudanjiang, Heilongjiang, China | Haomiao Zhang | 2007/7/25-08/05 |  |
| *Paracercion hieroglyphicum* | 116.365640 | 28.946407 | 1 | Nanchang, Jiangxi, China | Xin Yu | 2004/6/12 |  |
| *Paracercion hieroglyphicum* | 117.352678 | 38.994944 | 4 | Jinnan District, Tianjin, China | Xin Yu | 2016/7/27 |  |
| *Paracercion hieroglyphicum* | 117.178400 | 36.067000 | 9 | Taian, Shandong, China | Junli Xue | 2015/8/17 | 106 |
| *Paracercion hieroglyphicum* | 117.330568 | 39.006486 | 7 | Tianjin University, Tianjin, China | Xin Ning | 2015/6/13 |  |
| *Paracercion hieroglyphicum* | 117.401715 | 38.726810 | 1 | Binhai New Area, Tianjin, China | Teng Li | 2013/7/11 |  |
| *Paracercion hieroglyphicum* | 117.174454 | 39.094527 | 1 | Water park, Tianjin, China | Xin Yu | 2013/6/16 |  |
| *Paracercion hieroglyphicum* | 114.933203 | 40.687831 | 2 | Zhangjiakou, Hebei, China | Xin Yu | 2006/7/2 |  |
| *Paracercion melanotum* | 102.484409 | 24.925842 | 1 | Anning, Yunnan, China | Jin Chen | 2012/6/4 |  |
| *Paracercion melanotum* | 103.421292 | 24.714277 | 34 | Changhu town, Shilin, Yunnan, China | Xin Ning | 2016/6/30 |  |
| *Paracercion melanotum* | 100.191004 | 25.792294 | 3 | Dali, Yunnan, China | Xin Yu | 2006/8/18 | 1970 |
| *Paracercion melanotum* | 104.486719 | 29.593688 | 1 | Fu county, Zhaotong, Yunnan, China | Junli Xue | 2016/7/5 |  |
| *Paracercion melanotum* | 117.352678 | 38.994944 | 2 | Jinnan District, Tianjin, China | Xin Yu | 2016/7/27 |  |
| *Paracercion melanotum* | 117.178400 | 36.067000 | 9 | Taian, Shandong, China | Junli Xue | 2015/8/17 | 106 |
| *Paracercion melanotum* | 117.330568 | 39.006486 | 18 | Tianjin University, Tianjin, China | Xin Ning | 2015/6/13 |  |
| *Paracercion melanotum* | 117.329924 | 38.960660 | 3 | Binhai New Area, Tianjin, China | Teng Li | 2013/7/8 | 30 |
| *Paracercion melanotum* | 114.430833 | 30.546389 | 1 | Wuhan, Hubei, China | Xin Ning | 20140827 | 34 |
| *Paracercion melanotum* | 122.176771 | 30.175634 | 1 | Zhoushan, Zhejiang, China | Xin Yu | 2016/8/5 | 41 |
| *Paracercion plagiosum* | 123.019700 | 46.289000 | 1 | Hinggan League, Inner Mongolia, China | Xiurong Li & Yahui Zhen | 2014/7/23 | 140.5 |
| *Paracercion plagiosum* | 121.554198 | 41.003099 | 1 | Jinzhou, Liaoning, China | Xin Yu | 2008/7/25 | 6 |
| *Paracercion plagiosum* | 132.355970 | 45.467049 | 3 | Mishan, Heilongjiang, China | Xin Ning | 2016/8/19 |  |
| *Paracercion plagiosum* | 129.138056 | 44.166678 | 1 | Mudanjiang, Heilongjiang, China | Xin Ning | 2016/8/7 |  |
| *Paracercion plagiosum* | 117.182094 | 39.109420 | 5 | Nankai District, Tianjin, China | Haoyang Wu | 2012/6/12 |  |
| *Paracercion sieboldii* | 137.061340 | 36.851274 | 3 | Mukugahara, Fuchu, Toyama, Japan | Hiroyuki Futahashi | 2015/6/18 |  |
| *Paracercion sieboldii* | 139.982240 | 36.926943 | 2 | Wadai, Tsukuba, Ibaraki, Japan. | Ryo Futahashi | 2015/7/13 |  |
| *Paracercion v-nigrum* | 115.588651 | 39.646168 | 1 | Beijing, China | Xin Yu | 2008/ |  |
| *Paracercion v-nigrum* | 104.123436 | 30.630242 | 2 | Chengdu, Sichuan, China | Xin Yu | 2011/8/2 |  |
| *Paracercion v-nigrum* | 103.470808 | 29.577616 | 2 | Emeishan, Sichuan, China | Bingjiao Sun | 2015/7/22 | 450 |
| *Paracercion v-nigrum* | 108.056000 | 25.148000 | 1 | Hechi, Guangxi, China | Xin Yu | 2015/7/21 | 255 |
| *Paracercion v-nigrum* | 113.532883 | 35.447308 | 1 | Huixian, Henan, China | Danli Zhang | 2013/7/18 | 577 |
| *Paracercion v-nigrum* | 126.599520 | 43.865070 | 6 | Jilin, Jilin, China | Hongguang Jin | 2017/6/27 |  |
| *Paracercion v-nigrum* | 117.545935 | 40.184850 | 1 | Jixian County, Tianjin, China | Xin Yu | 2014/6/23 |  |
| *Paracercion v-nigrum* | 108.035031 | 25.235458 | 1 | Libo County, Guizhou, China | Xin Yu | 2010/6/13 |  |
| *Paracercion v-nigrum* | 107.942616 | 25.316544 | 1 | Libo County, Guizhou, China | Xin Yu | 2010/6/12 |  |
| *Paracercion v-nigrum* | 107.942616 | 25.316544 | 1 | Libo County, Guizhou, China | Xin Yu | 2010/6/12 | 783 |
| *Paracercion v-nigrum* | 107.899500 | 25.252000 | 2 | Libo County, Guizhou, China | Xin Ning | 2015/7/24-26 |  |
| *Paracercion v-nigrum* | 107.905500 | 25.283700 | 1 | Libo County, Guizhou, China | Junli Xue | 2015/7/24-26 | 776 |
| *Paracercion v-nigrum* | 107.905200 | 25.269800 | 3 | Libo County, Guizhou, China | Junli Xue | 2015/7/24-26 |  |
| *Paracercion v-nigrum* | 107.899500 | 25.252000 | 10 | Libo County, Guizhou, China | Xin Ning | 2015/7/24-26 |  |
| *Paracercion v-nigrum* | 107.899500 | 25.252000 | 7 | Libo County, Guizhou, China | Junli Xue | 2015/7/24-26 | 600 |
| *Paracercion v-nigrum* | 107.896200 | 25.251700 | 2 | Libo County, Guizhou, China | Junli Xue | 2015/7/24-26 | 600 |
| *Paracercion v-nigrum* | 107.896200 | 25.251700 | 1 | Libo County, Guizhou, China | Xin Yu | 2015/7/24-26 | 783 |
| *Paracercion v-nigrum* | 107.894000 | 25.252000 | 3 | Libo County, Guizhou, China | Xin Yu | 2015/7/24-26 | 787 |
| *Paracercion v-nigrum* | 107.905000 | 25.281000 | 2 | Libo County, Guizhou, China | Xin Yu | 2015/7/24-26 | 783 |
| *Paracercion v-nigrum* | 107.894000 | 25.252000 | 6 | Libo County, Guizhou, China | Xin Yu | 2015/7/24-26 |  |
| *Paracercion v-nigrum* | 107.894000 | 25.252000 | 9 | Libo County, Guizhou, China | Xin Ning | 2015/7/24-26 |  |
| *Paracercion v-nigrum* | 107.894000 | 25.252000 | 1 | Libo County, Guizhou, China | Junli Xue | 2017/7/4 | 764 |
| *Paracercion v-nigrum* | 111.657600 | 33.940800 | 9 | Luoyang, Henan, China | Yao Ji | 2013/7/26 |  |
| *Paracercion v-nigrum* | 103.237559 | 29.724938 | 8 | Meishan, Sichuan, China | Xin Yu | 2016/8/7 |  |
| *Paracercion v-nigrum* | 103.760624 | 30.096270 | 1 | Meishan, Sichuan, China | Xin Yu | 2009/5/23 |  |
| *Paracercion v-nigrum* | 129.138056 | 44.166678 | 9 | Mudanjiang, Heilongjiang, China | Xin Ning | 2016/8/8 |  |
| *Paracercion v-nigrum* | 128.919244 | 43.883723 | 1 | Ningan, Heilongjiang, China | Xin Yu | 2015/07/31-08/02 | 760 |
| *Paracercion v-nigrum* | 108.340300 | 26.341700 | 17 | Rongjiang County, Guizhou, China | Xin Ning | 2015/07/31-08/02 | 760 |
| *Paracercion v-nigrum* | 108.340300 | 26.341700 | 2 | Rongjiang County, Guizhou, China | Junli Xue | 2015/07/31-08/02 | 616 |
| *Paracercion v-nigrum* | 108.340300 | 26.341700 | 2 | Rongjiang County, Guizhou, China | Xin Yu | 2012/8/3 |  |
| *Paracercion v-nigrum* | 118.190411 | 40.400091 | 2 | Tangshan, Hebei, China | Teng Li | 2015/8/11 |  |
| *Paracercion v-nigrum* | 110.262200 | 36.752700 | 5 | Yanchuan County, Shanxi, China | Xin Ning | 2016/7/4 |  |
| *Paracercion v-nigrum* | 104.260489 | 27.768680 | 12 | Yiliang County, Yunnan, China | Xin Ning | 2016/7/4 |  |
| *Paracercion v-nigrum* | 104.260489 | 27.768680 | 9 | Yiliang County, Yunnan, China | Junli Xue | 2016/7/4 |  |
| *Paracercion v-nigrum* | 104.260489 | 27.768680 | 1 | Yiliang County, Yunnan, China | Xin Ning | 2016/8/2 | 778 |
| *Paracercion v-nigrum* | 121.077046 | 29.745519 | 4 | Yuyao, Zhejiang, China | Xin Yu | 2010/ |  |
| *Paracercion v-nigrum* | 107.296249 | 28.733658 | 1 | Zunyi, Guizhou, China | Xin Yu et al. | 2010/6/5 | 1500 |
| *Paracercion v-nigrum* | 107.296249 | 28.733658 | 1 | Zunyi, Guizhou, China | Kai Dang | 2010/6/4 |  |
| *Paracercion v-nigrum* | 107.296249 | 28.733658 | 1 | Zunyi, Guizhou, China | Xin Yu |  |  |
| *Paracercion calamorum* AB706626 |  |  |  |  |  |  |  |
| *Paracercion calamorum* AB706627 |  |  |  |  |  |  |  |
| *Paracercion hieroglyphicum* AB706628 |  |  |  |  |  |  |  |
| *Paracercion hieroglyphicum* AB706630 |  |  |  |  |  |  |  |
| *Paracercion hieroglyphicum* AB706631 |  |  |  |  |  |  |  |
| *Paracercion hieroglyphicum* AJ488551 |  |  |  |  |  |  |  |
| *Paracercion melanotum* AB706633 |  |  |  |  |  |  |  |
| *Paracercion melanotum* AB706635 |  |  |  |  |  |  |  |
| *Paracercion melanotum* AB706636 |  |  |  |  |  |  |  |
| *Paracercion plagiosum* AB706637 |  |  |  |  |  |  |  |
| *Paracercion plagiosum* AJ488552 |  |  |  |  |  |  |  |
| *Paracercion sieboldii* AB706638 |  |  |  |  |  |  |  |
| *Paracercion sieboldii* AB706639 |  |  |  |  |  |  |  |
| *Paracercion sieboldii* AB706640 |  |  |  |  |  |  |  |
| *Paracercion sieboldii* AJ621056 |  |  |  |  |  |  |  |
| *Paracercion v-nigrum* AJ621057 |  |  |  |  |  |  |  |
| *Coenagrion ornatum* AJ621059 |  |  |  |  |  |  |  |
| *Coenagrion puella* AJ488546 |  |  |  |  |  |  |  |
| *Coenagrion pulchellum* AJ488547 |  |  |  |  |  |  |  |
| *Erythromma najas* AJ621054 |  |  |  |  |  |  |  |
| *Erythromma najas* AJ621055 |  |  |  |  |  |  |  |
| *Erythromma viridulum* AJ621058 |  |  |  |  |  |  |  |
| *Ischnura graellsi* AJ488545 |  |  |  |  |  |  |  |

Table S2 Average intraspecific and interspecific genetic distance of the COI gene

|  | *P. ambiguum* | *P. barbatum* | *P. calamorum* | *P. dorothea* | *P. hieroglyphicum* | *P. melanotum* | *P. plagiosum* | *P. sieboldii* | *P. v.nigrum* |
| --- | --- | --- | --- | --- | --- | --- | --- | --- | --- |
| *P. ambiguum* | **0.0038** |  |  |  |  |  |  |  |  |
| *P. barbatum* | 0.1435 | **0.0008** |  |  |  |  |  |  |  |
| *P. calamorum* | 0.1530 | 0.0738 | **0.0013** |  |  |  |  |  |  |
| *P. dorothea* | 0.1408 | 0.1327 | 0.1494 | **0.0003** |  |  |  |  |  |
| *P. hieroglyphicum* | 0.1598 | 0.1003 | 0.0942 | 0.1301 | **0.0068** |  |  |  |  |
| *P. melanotum* | 0.1601 | 0.0995 | 0.0943 | 0.1281 | 0.0069 | **0.0049** |  |  |  |
| *P. plagiosum* | 0.1434 | 0.1211 | 0.1312 | 0.1382 | 0.1244 | 0.1245 | **0.0005** |  |  |
| *P. sieboldii* | 0.1526 | 0.0331 | 0.0805 | 0.1422 | 0.0991 | 0.0982 | 0.1173 | **0.0011** |  |
| *P. v-nigrum* | 0.1446 | 0.0049 | 0.0742 | 0.1344 | 0.1010 | 0.1001 | 0.1220 | 0.0346 | **0.0067** |

Notes: The average intraspecific K2P distances were shown in bold.

Table S3 Average intraspecific and interspecific genetic distance of the ITS marker

|  | *P. ambiguum* | *P. barbatum* | *P. calamorum* | *P. dorothea* | *P. hieroglyphicum* | *P. melanotum* | *P. plagiosum* | *P. sieboldii* | *P. v.nigrum* |
| --- | --- | --- | --- | --- | --- | --- | --- | --- | --- |
| *P. ambiguum* | **0.0000** |  |  |  |  |  |  |  |  |
| *P. barbatum* | 0.1515 | **0.0301** |  |  |  |  |  |  |  |
| *P. calamorum* | 0.1503 | 0.0872 | **0.0220** |  |  |  |  |  |  |
| *P. dorothea* | 0.1026 | 0.1367 | 0.1260 | **0.0011** |  |  |  |  |  |
| *P. hieroglyphicum* | 0.1427 | 0.0855 | 0.0816 | 0.1434 | **0.0006** |  |  |  |  |
| *P. melanotum* | 0.1590 | 0.0648 | 0.1063 | 0.1526 | 0.1011 | **0.0101** |  |  |  |
| *P. plagiosum* | 0.1257 | 0.1366 | 0.1207 | 0.0981 | 0.1307 | 0.1524 | **0.0060** |  |  |
| *P. sieboldii* | 0.1544 | 0.0473 | 0.0777 | 0.1191 | 0.0833 | 0.0772 | 0.1287 | **0.0131** |  |
| *P. v-nigrum* | 0.1527 | 0.0474 | 0.0739 | 0.1302 | 0.0763 | 0.0692 | 0.1280 | 0.0198 | **0.0189** |

Notes: The average intraspecific K2P distances were shown in bold.

Table S4 Detailed result of the ABGD analysis (P = 0.0129)

| Marker | Group | Morphospecies | Number |
| --- | --- | --- | --- |
| COI | Group1 | *P. barbatum* & *P. sieboldii* & *P. v-nigrum* | 188 |
|  | Group2 | *P. calamorum* | 31 |
|  | Group3 | *P. dorothea* | 6 |
|  | Group4 | *P. hieroglyphicum* & *P. melanotum* | 110 |
|  | Group5 | *P. plagiosum* | 11 |
|  | Group6 | *P. ambiguum* | 2 |
| ITS | Group1 | *P. ambiguum* | 2 |
|  | Group2 | *P. barbatum* & *P. sieboldii* & *P. v-nigrum* | 24 |
|  | Group3 | *P. barbatum* & *P. melanotum* | 12 |
|  | Group4 | *P. calamorum* | 5 |
|  | Group5 | *P. dorothea* | 3 |
|  | Group6 | *P. hieroglyphicum* | 14 |
|  | Group7 | *P. plagiosum* | 6 |

Table S5 Detailed result of the clustering analysis (Threshold = 0.03)

| Marker | Cluster | Morphospecies | Number |
| --- | --- | --- | --- |
| COI | Cluster1 | *P. ambiguum* | 2 |
|  | Cluster2 | *P. barbatum* & *P. v-nigrum* | 183 |
|  | Cluster3 | *P. calamorum* | 31 |
|  | Cluster4 | *P. dorothea* | 6 |
|  | Cluster5 | *P. hieroglyphicum* & *P. melanotum* | 110 |
|  | Cluster6 | *P. plagiosum* | 11 |
|  | Cluster7 | *P. sieboldii* | 5 |
| ITS | Cluster1 | *P. ambiguum* | 2 |
|  | Cluster2 | *P. barbatum* & *P. sieboldii* & *P. v-nigrum* | 24 |
|  | Cluster3 | *P. barbatum* & *P. melanotum* | 12 |
|  | Cluster4 | *P. calamorum* | 5 |
|  | Cluster5 | *P. dorothea* | 3 |
|  | Cluster6 | *P. hieroglyphicum* | 14 |
|  | Cluster7 | *P. plagiosum* | 6 |

Table S6 Detailed result of the GMYC analysis

| Marker | GMYC_Speices | Number of haplotype | Morphospecies |
| --- | --- | --- | --- |
| COI | 1 | 2 | *P. ambiguum* |
|  | 2 | 11 | *P. calamorum* |
|  | 3 | 70 | *P. barbatum* & *P. v-nigrum* |
|  | 4 | 3 | *P. sieboldii* |
|  | 5 | 55 | *P. hieroglyphicum* & *P. melanotum* |
|  | 6 | 2 | *P. plagiosum* |
|  | 7 | 2 | *P. dorothea* |
| ITS | 1 | 2 | *P. dorothea* |
|  | 2 | 4 | *P. plagiosum* |
|  | 3 | 2 | *P. calamorum* |
|  | 4 | 3 | *P. calamorum* |
|  | 5 | 6 | *P. hieroglyphicum* |
|  | 6 | 10 | *P. barbatum* & *P. melanotum* |
|  | 7 | 4 | *P. barbatum* |
|  | 8 | 11 | *P. sieboldii* & *P. v-nigrum* |
|  | 9 | 5 | *P. sieboldii* & *P. v-nigrum* |
|  | 10 | 1 | *P. ambiguum* |
|  | 11 | 1 | *P. barbatum* |
|  | 12 | 1 | *P. barbatum* |

Table S7 Detailed result of the bPTP analysis

| Marker | bPTP_Speices | Support | Number of haplotype | Morphospecies |
| --- | --- | --- | --- | --- |
| COI | Species 1 | 0.788 | 2 | *P ambiguum* |
|  | Species 2 | 0.941 | 2 | *P dorothea* |
|  | Species 3 | 0.943 | 2 | *P plagiosum* |
|  | Species 4 | 0.988 | 55 | *P hieroglyphicum* & *P melanotum* |
|  | Species 5 | 0.785 | 11 | *P calamorum* |
|  | Species 6 | 0.842 | 70 | *P barbatum* & *P v-nigrum* |
|  | Species 7 | 0.931 | 3 | *P sieboldii* |
| ITS | Species 1 | 1 | 1 | *P ambiguum* |
|  | Species 2 | 0.809 | 2 | *P dorothea* |
|  | Species 3 | 0.827 | 4 | *P plagiosum* |
|  | Species 4 | 0.73 | 10 | *P barbatum* & *P melanotum* |
|  | Species 5 | 0.986 | 1 | *P barbatum* |
|  | Species 6 | 0.984 | 1 | *P barbatum* |
|  | Species 7 | 0.62 | 4 | *P barbatum* |
|  | Species 8 | 0.368 | 16 | *P sieboldii* & *P v-nigrum* |
|  | Species 9 | 0.26 | 5 | *P calamorum* |
|  | Species 10 | 0.47 | 6 | *P hieroglyphicum* |
